# Working Memory-Related Prefrontal Hemodynamic Responses in University Students: A Correlation Study of Subjective Well-Being and Lifestyle Habits

**DOI:** 10.3389/fnbeh.2019.00213

**Published:** 2019-09-13

**Authors:** Yoichi Kawaike, Junko Nagata, Tamotsu Furuya, Chihaya Koriyama, Masayuki Nakamura, Akira Sano

**Affiliations:** ^1^Health Service Center, Kagoshima University, Kagoshima, Japan; ^2^Department of Psychiatry, Kagoshima University Graduate School of Medical and Dental Sciences, Kagoshima, Japan; ^3^Computing and Communication Center, Kagoshima University, Kagoshima, Japan; ^4^Department of Epidemiology and Preventive Medicine, Kagoshima University Graduate School of Medical and Dental Sciences, Kagoshima, Japan

**Keywords:** wearable near-infrared spectroscopy, working memory, subjective well-being, lifestyle, university students, mental health

## Abstract

Identification of social risk factors and the promotion of stress coping mechanisms and mental resilience are topics of interest in the field of mental health. The relationships between risk- or tolerability-associated factors and task-related hemodynamic responses in the prefrontal cortex (PFC) in adolescents may have important implications for mental health challenges. The purpose of this study was to investigate the relationship between task-related PFC hemodynamic activities and subjective well-being or lifestyle habits using wearable near-infrared spectroscopy (NIRS). In this study, after sample refinement to reduce heterogeneity, 20 university students were included in verbal working memory (VWM) task analyses and 21 were included in spatial working memory (SWM) task analyses. The task-related hemodynamic responses were detected using wearable NIRS. To assess the risk- or tolerability-associated factors, the levels of positive and negative affect were assessed using the Subjective Well-Being Inventory (SUBI) and lifestyle habits (such as gaming) were evaluated using a nine-item questionnaire. There was a positive correlation between SUBI positive affect and VWM task-related oxy-hemoglobin signal changes in the right dorsolateral PFC (DLPFC), underlining the significance of subjective well-being as an important independent emotional domain and suggesting the possibility of the differential objective evaluations of subjective well-being in the right PFC. Negative correlations between PFC activities during both VWM and SWM tasks at the left DLPFC and the number of game playing days in 1 week were also statistically significant, suggesting the presence of modality-non-specific hemodynamic regulation by habitual game playing. Each correlation was still robust after the elimination of major confounding impacts. Although further replication studies are warranted to confirm these preliminary results, this investigation of the relationship between task-related PFC hemodynamic activities and emotional domains or lifestyle habits might have clinical significance with regard to primary prevention of mental health issues in university students. To our knowledge, this is the first demonstration of these relationships with the use of wearable NIRS, which enables measurement under near natural conditions and is easy to use in schools or workplaces.

## Introduction

Identification of modifiable social risk factors across the lifespan and the promotion of prevention are topics of interest in the field of global mental health (Collins et al., 2011). In Japan, a nation-wide policy for psychosocial stress screening in the workplace was launched in 2015. Modification of problematic working environments can be made available for workers, and it was officially announced that workers in stressful environments should be aware of their stress level and enhance their stress coping mechanisms and mental resilience (Kawakami and Tsutsumi, 2016). However, factors that promote resilience and prevent mental disorders in persons at extreme social disadvantage remain to be fully elucidated, and no biological risk factor or marker has been validated for clinical use across the lifespan (Collins et al., 2011). Furthermore, our understanding of the roles of psychological components in psychopathological processes remains quite rudimentary (Clark et al., 2017). Because many lifestyle-related attitudes and habits can be formed in late adolescence and assessing well-being may be beneficial in predicting future depression risk (Sawyer et al., 2012; Grant et al., 2013), neurological findings associated with lifestyle habits and states of subjective well-being in university students may provide an important foundation for future investigations.

Subjective well-being is a complex characteristic of positive psychological functioning that captures an individual’s level of positive affect, life satisfaction, and sense of achievement (Sell and Nagpal, 1992). Longitudinal studies have demonstrated that subjective well-being or change in the well-being level are predictive of mental disorders, particularly stress-related disorders (Keyes et al., 2010; Grant et al., 2013). Although subjective well-being and subjective ill-being (negative affect) sometimes measure different ends of a single continuum, the relationship between them is not necessarily a simple inverse correlation and the relationship between different modalities (e.g., positive affect and negative mood) is more complex. It has been demonstrated that positive affect and negative affect are significantly related among patient groups with high subjective ill-being, but there was no correlation among healthy controls with a low subjective ill-being (Ono et al., 1995). Well-being scales have an extraversion component that is not shared by negative affect (Jorm and Duncan-Jones, 1990), a decrease in negative emotions does not necessarily promote an increase in positive emotions, and the predictive implication of the presence of positive emotions is different from that of the absence of negative emotions (Grant et al., 2013). Therefore, it may be important to assess both ends of the complex well-being dimension. The Subjective Well-Being Inventory (SUBI) is a questionnaire developed by the World Health Organization (WHO) on the basis of such perspectives (Sell and Nagpal, 1992). It aims to evaluate positive affect and negative affect from psychological, physical, and social aspects. In the SUBI, persons in a high positive affect state experience satisfaction, self-confidence, and connection with their surroundings, and they are considered to be in a state of high resistance to stress, while persons in a high negative affect state are considered to be in a tired state with regard to both physical and mental conditions. The Japanese version of the SUBI has been established by Ono et al. (1995) and the validity has been verified (Tonan et al., 1995).

The prefrontal cortex (PFC) is a recurrent site of integration between human affect and cognition, and significant complex interactions between PFC functions and human affect have been demonstrated in neuroimaging studies (Mitchell and Phillips, 2007). Because minor fluctuations in affect or mood can influence such PFC functions (Mitchell and Phillips, 2007), the simplification of the measurement process and experimental environments may be quite important for the evaluation of hemodynamic responses. Near-infrared spectroscopy (NIRS) is a non-invasive and low-constraint neuroimaging technique and is available as a wearable device, which can minimize the artificial impact on the individual affect. Statistically significant negative correlations between PFC activities during a verbal working memory (VWM) task and a negative mood score have been demonstrated using NIRS in healthy participants, suggesting the significance of NIRS-measured PFC activity as a possible biomarker of the state of affect in healthy individuals (Aoki et al., 2011, 2013; Sato et al., 2011, 2014). Therefore, the first aim of this study was to investigate the relationships between SUBI scores and PFC activities during working memory tasks using a wearable NIRS device. Clarifying the relationships between PFC activities and an individual’s affect including positive and negative affect (i.e., well-being) might lead to the elucidation of the neurological processes of resilience and vulnerability to mental disorders. NIRS evaluations can be used to consolidate the diagnosis of depression and the task-related PFC response may have clinical implications in some mental disorders (Takizawa et al., 2008, 2014; Suda et al., 2010). In the hierarchical complex interactions among psychological well-being, subjective well-being, and the manifestation of psychiatric conditions (Burns et al., 2011), NIRS evaluations might provide a useful objective marker for the level of subjective well-being.

It has been reported in many studies that excessive drinking and abuse of the internet, smartphones, and video games undermine mental functions directly or indirectly (Griffiths and Meredith, 2009; Lam and Peng, 2010; Kim et al., 2018; Stankewicz and Salen, 2018; Stockdale and Coyne, 2018). However, physical exercise is associated with resilience to depression and tolerance to stressful episodes (Stanton and Reaburn, 2014; Harvey et al., 2018). As the second aim of this study, the relationships between individual lifestyle habits and PFC activities during working memory tasks were investigated. In order to consider the possibility of primary prevention, the correlations between lifestyle habits and the subjective well-being were also assessed.

## Materials and Methods

This study was approved by the Institutional Review Board Ethics Committee of the Health Service Center, Kagoshima University (accession no.: H26-4-1). Written informed consent was obtained from all participants after the purpose, procedure, risks, benefits, personal information management, and voluntary nature of participation had been explained to them. In the analysis processes, all data were anonymized and the researchers were blinded to the links between the data and personal information.

### Participants

**Figure 1:**
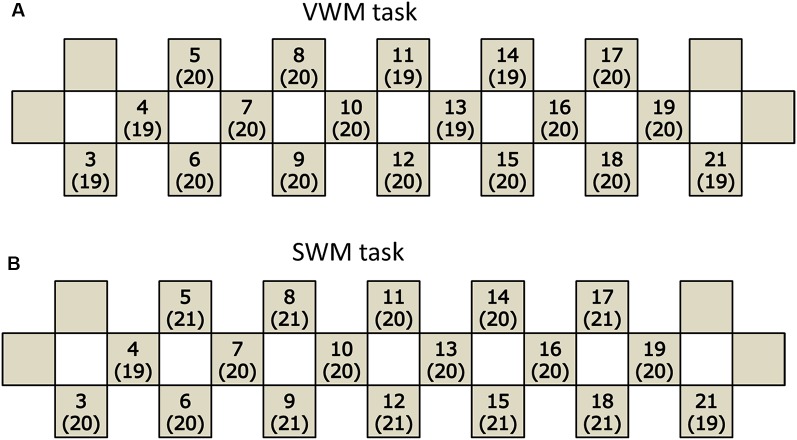
Participants included in the analysis of each task. The number of participants remaining in the final analysis for each channel in the verbal working memory (VWM) task **(A)** and spatial working memory (SWM) task **(B)**. Each channel is indicated by a square with the channel number and the sample number in parentheses.

A total of 28 participants were initially enrolled. The participants included in the final analysis were selected using the criteria described below (handedness, taking medication, task performance). The recruitment was sequential and the participants were all Japanese-speaking and undergraduate or graduate students at Kagoshima University. Handedness was assessed using the Edinburgh Handedness Inventory (EHI; Oldfield, 1971) in association with the possible lateralization of brain functions. Two women whose handedness was determined to be not right-handed were excluded from the subsequent data analysis because the sample was too small for lateralization consideration. Three other women were also excluded because they were taking oral medicine. In the remaining 23 participants, two women exhibited poor behavioral performance (<60% correct responses for each task) in both the VWM and spatial working memory (SWM) tasks, and another woman exhibited poor behavioral performance only in VWM task. Because such poor behavioral performance might be an accidental outcome of the participant’s transient condition or may be subject to the artificial effects of experimental environments, the participants were excluded from the subsequent data analyses. Finally, 20 participants (12 females and 8 males) were included in the VWM task analyses. In the VWM task analyses, the mean age was 23.4 years (standard deviation, SD = 1.7) and the age range was 21–27 years. In the SWM task analyses, 21 participants (13 females and eight males) were included. In the SWM task analyses, the mean age was 23.4 years (SD = 1.6) and the age range was 21–27 years. In addition, because the detection of near-infrared spectrum light from the skin surface is critical in NIRS, the responses were not necessarily fully detected due to potential hair or motion artifacts. Therefore, the sample numbers varied for each probe channel (Figures 1A,B). The range of sample numbers was 19–20 and 19–21 for the VWM and SWM task analyses, respectively. All analyzed participants reported that they were not currently experiencing overt disease or injury on the health condition questionnaire (described below). There was no apparent heterogeneity in ethnic backgrounds and educational achievement among the participants. No participants experienced lack of sleep the night before NIRS measurement.

### Subjective Well-Being, Health Conditions, and Lifestyle Habits

Subjective well-being was assessed using the Japanese version of the SUBI (Tonan et al., 1995). The SUBI is a 40-item questionnaire with two axes; positive affect (subjective well-being) and negative affect (subjective ill-being; Sell and Nagpal, 1992). Positive affect is comprised of six subscales; “general well-being” (positive affect), “expectation-achievement congruence,” “confidence in coping,” transcendence, “family group support,” and “social support.” Negative affect has four subscales; “inadequate mental mastery,” “perceived ill-health,” “deficiency in social contacts,” and “general well-being” (negative affect). The “primary group concern” subscale is included in both positive and negative affect axes. The score for each question ranges from 1 to 3 and a higher score indicates a better state. Health conditions and lifestyle habits were evaluated using a nine-item questionnaire. In the questionnaire, medication use, present illness or history of overt disease or injury, drinking habit (the number of drinking days per week), smoking habit (the number of cigarettes smoked in 1 day), internet use in the past 6 months (average hours per day), smartphone use in the past 6 months (average hours per day), gaming habit in the past 6 months (average days in 1 week and average hours per day for PC games, smartphone games, mobile games, handheld game consoles, and video games), and frequency of physical exercise in the past 6 months (average number of days of exercise per week) were assessed. When the participants reported “2–3 h” or “3–5 days,” the average values were used for the analyses. Since there was only one participant who had a smoking habit among all participants, smoking habit was excluded from the statistical analyses.

### Working Memory Tasks

**Figure 2:**
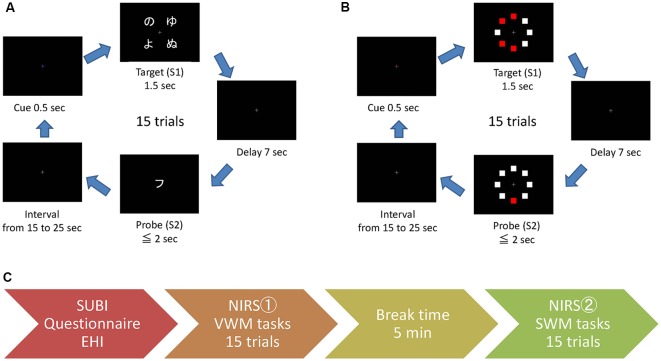
The sequence of working memory tasks and experimental procedure. For the VWM task **(A)**, four Hiragana characters were presented as the target (S1) and a Katakana character was presented as the probe (query) stimulus (S2). For the SWM task **(B)**, eight squares including four red squares were presented as the target (S1) and one red square and seven white squares were presented as the probe (query; S2). The experimental procedure is outlined in the aligned feathered arrow shapes **(C)**. SUBI, Subjective Well-Being Inventory; NIRS, near-infrared spectroscopy.

The temporary retention of verbal or spatial information was induced by a computational sequence of the target information along with a query regarding this information (a delayed match-to-sample task). The signal-averaging of the task-related NIRS responses was validated as a biomarker of PFC working memory functions, and the tasks used in this study were essentially the same as those in previous NIRS studies (Aoki et al., 2011, 2013; Sato et al., 2011, 2014; Watanabe et al., 2016). The tasks were presented to the participants using a monitor and speakers. The responses to queries were submitted by the participant *via* a handheld gamepad (controller). The working memory tasks, VWM and SWM, were controlled by a laptop computer that was connected and synchronized with the other laptop for NIRS analyses. In each trial of both tasks, the target stimulus (S1) was visualized for 1.5 s, and following a 7.0 s delay, the probe (query) stimulus (S2) was exhibited for 2.0 s or until the response submission (Figures 2A,B). There was a random interval from 15 s to 25 s between the end of S2 and the onset of S1 of the next trial. During the interval and delay, a small gray cross was presented at the center of the monitor as the gaze target. As a visual cue for the next trial, the color of the cross was changed to blue for the VWM task or red for the SWM task at 0.5 s prior to the onset of S1. As auditory cues, 1,000 and 800 Hz beeps were presented for 0.1 s at the onset of the visual cue and S2, respectively.

Because there are two types of Japanese syllable-characters (Hiragana and Katakana) that can be identified by the individual syllable, the type change between S1 and S2 compels the participant to retain the verbal (letter) information of S1 for the response to the S2 query in the VWM task. Four Hiragana characters were presented as S1, and a Katakana character was presented as S2 (Figure 2A). The participant was asked to determine whether the S2 character corresponded to one of the S1 characters in the VWM task. In the SWM task, eight squares (four white and four red squares) were randomly arranged in a circle as S1, and the same eight square arrangement consisting of one red and seven white squares was presented as S2 (Figure 2B). Participants were asked to determine whether the location of the red square presented as S2 corresponded to one of the four locations of red squares in S1. For each task, the accuracy of the responses to S2 queries and the delay time between the onset of S2 and the response were evaluated as the task performance.

### Procedure

The procedure for this experiment is illustrated in Figure 2C. Prior to NIRS measurement, participants completed the EHI, the questionnaire regarding health conditions and lifestyle habits, and the SUBI. The participants then moved to a dimly lit, quiet, air-conditioned room for the task-related NIRS measurement. The probe unit was attached to the participant on a chair with a headrest, and a light-shielding sheet was fixed adjacent to the probe unit to reduce the influence of external light. The distance between the participant’s face and the monitor was approximately 50 cm. Participants were instructed to fix their head to the headrest and to maintain the direction of gaze at the center of the monitor (the cross target). There were no moving objects (or objects with numbers or letters) outwith the monitor in the participant’s visual field, and the examiner sat behind the participant. The task procedure was automatically explained using the monitor and speakers, and the participants then started performing the first task in their own time. All participants performed the VWM task session (15 trials) before the SWM task session (15 trials), with a 5 min interval between the sessions.

### NIRS Measurement

**Figure 3:**
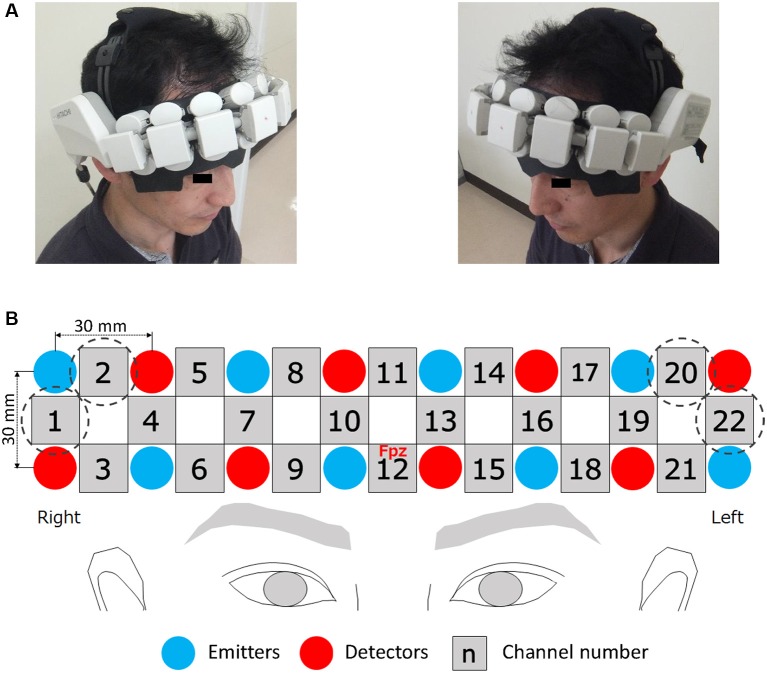
Wearable NIRS device. The attached probe unit (a wearable package as a headset) can fully cover the forehead **(A)**. In the probe unit, emitters and detectors are alternately arranged in two rows at an inter-probe distance of 30 mm **(B)**. The center channel was placed on the Fpz point. Channels surrounded by dotted circles were excluded from analysis. Written informed consent was obtained from this individual **(A)** for publication of this image.

A wearable NIRS system (WOT, Hitachi High-Technologies Company Limited, Japan) was used to measure PFC activity. The attached probe unit (a wearable package as a headset), composed of eight emitters and eight detectors, can fully cover the participant’s forehead (Figure 3A). The detectors sent the detected signals to the processing unit (a portable control box), and these data were computationally processed. The processed signals were recorded on the laptop computer for NIRS measurement *via* the local wireless network. In the probe unit, the emitters and detectors are alternately arranged in two rows at an inter-probe distance of 3 cm (Figure 3B). Because the processed signal originates from an emitter probe and an adjacent detector probe corresponds to the PFC activity in the region between these two probes, this system has 22 measurement points (22 channels). Channels 1, 2, 20, and 22 were located in regions covered in hair in all participants and the detection of PFC activity at these channels was near impossible. Therefore, data from a total of 18 channels were available in this study (Figure 3B). The emitters provided two types of near-infrared light with wavelengths of 705 and 830 nm and the probes detected the reflected light every 0.2 s. The relative changes in oxygenated hemoglobin (oxy-Hb) and deoxygenated Hb (deoxy-Hb) were calculated on the basis of the modified Beer–Lambert law (Delpy et al., 1988), using light signals at these two wavelengths. The bottom row of the probe unit was adjusted to correspond to the line of Fp1-Fp2 in the international 10-20 system, and its center (Channel 12) was placed at Fpz, as illustrated in Figure 3B. To estimate the location of the channels, a reported 3-D topographical map, which was introduced using the probabilistic registration method, was used as a reference (Aoki et al., 2011).

### Data Processing and Statistical Analysis

A plug-in based application (Platform for Optical Topography Analysis Tools) developed by Hitachi ARL on MATLAB (The MathWorks, Inc., Natick, MA, USA; Sutoko et al., 2016) and a statistical software package (JMP, version 13.0.0; SAS Institute Inc., Arlington, VA, USA) were used for the statistical analyses. In this study, the relative signal changes in oxy-Hb were considered to be important, because oxy-Hb changes are assumed to reflect brain activity more directly than deoxy-Hb changes (Hoshi et al., 2001; Strangman et al., 2002). The data sequence for each channel was divided into 28.5 s task blocks. Each block consisted of a 4 s pre-task period, an 8.5 s task period (during the 1.5 s S1 presentation and the 7 s delay period), a 12 s recovery period (starting from the S2 onset), and 4 s post-task period. Motion artifacts were objectively detected as un-physiologically sharp noises with a relative gradient of 0.2 or more per 0.2 s and effectively excluded (Peña et al., 2003; Aoki et al., 2011). The noise-free data sequences were smoothed with a 5 s moving average and the baselines were corrected by linear regression based on the least-squares method using the data from the pre-task and post-task periods of the block (Aoki et al., 2013).

Signal-averaging was applied to depict the task-related oxygenation process at each channel across all available analyses (i.e., 21 in the VWM task and 20 in the SWM task). The noise-free and baseline-corrected data sequence were also utilized to evaluate the task-related oxy-Hb signal change. In each trial block of each participant, the PFC activation value was calculated (Aoki et al., 2011) to confirm the validity of the trial. The activation value was the difference between the mean oxy-Hb signal during the activation period and that during the pre-task period (zero). The duration of an activation period was 3.5 s, which started 5.0 s after S1 onset and ended at S2 onset in the trial block. Channels with invalid or missing activation peaks in more than eight trial blocks were excluded from the subsequent analyses. When the activation values were zero in all trial blocks, the participant was excluded from the channel analyses (one at channel 21 in the VWM task and one at channels 11 and 21 in the SWM task, respectively). Therefore, the sample numbers varied for each probe channel in this study, as described above. For correlation evaluation, the activation values were averaged at each channel in each participant (task-related oxy-Hb signal change). To statistically assess the significance of PFC activation, a *t*-test (one sample, one-tailed) was used to analyze the average task-related oxy-Hb signal changes across participants (Aoki et al., 2011, 2013; Sato et al., 2011, 2014). To examine the correlations between the task-related oxy-Hb signal changes and non-parametric ratings, Spearman’s rank correlation was used (Aoki et al., 2011, 2013; Sato et al., 2011, 2014). For lifestyle habits where the task was significantly related to oxy-Hb signal changes, a *t*-test (one-tailed) was used to confirm the difference in oxy-Hb signal change averages between students with the habit and those without the habit. The correlations among questionnaire scores, task performance (reaction time and accuracy), and age and the task-related oxy-Hb signal change were evaluated. In box-and-whisker plotting for the task-related oxy-Hb signal changes, cases whose values were outside the box edges by more than 1.5 times the interquartile range were objectively determined as outliers. The inter-relationships between questionnaire scores and age were investigated in the final group of valid participants. To evaluate sex differences in the task-related oxy-Hb signal change and questionnaire scores, the average across participants was compared between males and females for each task by Student’s *t*-test and Wilcoxon rank sum test. For the false discovery rate (FDR) correction for multiple comparisons among a total of 18 channels, the FDR threshold was set to 0.05 (Benjamini and Hochberg, 1995; Singh and Dan, 2006). In this article, the uncorrected *p* values are referred to as “*p*” and the corrected *p* values by the FDR method are denoted by “*q*.”

## Results

### The SUBI and the Other Conditions Including Lifestyle Scores

For the final group of 21 valid participants (eight males and 13 females), we calculated the mean and SD of the SUBI and lifestyle questionnaire scores. In the SUBI, the mean scores were 41.19 ± 5.99 for positive affect and 50.19 ± 4.71 for negative affect. In the lifestyle questionnaire, the mean scores were 0.79 ± 0.75 h/day and 3.33 ± 3.04 days/week for playing games, 1.14 ± 1.77 days/week for drinking, 3.10 ± 3.22 h/day for internet use, 2.62 ± 0.86 h/day for smartphone use, and 0.95 ± 1.63 h/week for physical exercise. Correlations between the SUBI and lifestyle questionnaire scores and age are presented in Table 1. Significant correlations were observed between the negative affect score (a lower score indicates higher intensity of negative affect) and internet use time (*r* = −0.44, *p* = 0.0450), between game playing time in 1 day (h/day) and in 1 week (days/week; *r* = 0.88, *p* < 0.0001), between days of drinking and days of gaming in 1 week (*r* = 0.52, *p* = 0.0167), and between the hours of smartphone use and hours of internet use in 1 day (*r* = 0.73, *p* = 0.0002). Although there were significant differences between sexes in the average smartphone use (h) per day (male: 2.06 ± 0.86, female: 2.96 ± 0.69, *z* = −2.18, *p* = 0.03) and the number of days of exercise in 1 week (male: 1.88 ± 1.96, female: 0.38 ± 1.12, *z* = −2.23, *p* = 0.03), no significant differences were observed between sexes in the other variables [SUBI positive affect score (*z* = −0.76, *p* = 0.45), SUBI negative affect score (*z* = 0.36, *p* = 0.72), number of drinking days in 1 week (*z* = 1.13, *p* = 0.26), average internet use hours per day (*z* = −1.07, *p* = 0.28), game playing days in 1 week (*z* = 1.08, *p* = 0.28), average game playing hours per day (*z* = 0.34, *p* = 0.74)].

**Table 1:** Correlation between the Subjective Well-Being Inventory (SUBI) score and lifestyle questionnaire.

Variables	Positive	Negative	Drinking	Game D	Game W	Internet	Smartphone	Exercise
Age	−0.36	0.21	−0.16	−0.10	−0.23	0.14	−0.02	0.22
Exercise	0.14	0.19	0.09	−0.03	0.00	−0.16	−0.38	
Smartphone	0.18	−0.21	−0.16	0.39	0.21	0.73**		
Internet	−0.16	−0.44*	0.00	0.42	0.30			
Game W	0.07	−0.39	0.52*	0.88**				
Game D	0.00	−0.37	0.33					
Drinking	0.17	−0.19						
Negative	0.37							

Note: Positive indicates the Subjective Well-Being Inventory (SUBI) positive affect score, *Negative* indicates the SUBI negative affect score, *Drinking* indicates the number of drinking days in 1 week, *Game D* indicates the average gaming hours per day, *Game W* indicates the average number of gaming days in 1 week, *Internet* indicates the average internet use hours per day, *Smartphone* indicates the average smartphone use hours per day, *Exercise* indicates the number of days of exercise in 1 week. All values are Spearman’s rank correlation coefficients for the final group of 21 valid participants in both tasks (*P < 0.05 uncorrected, **P < 0.01 uncorrected).

### PFC Activities

**Figure 4:**
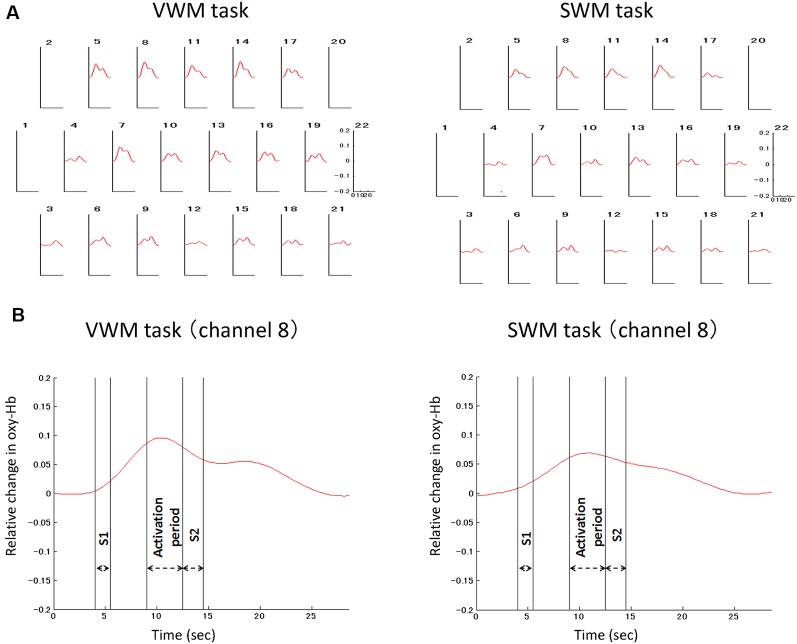
Grand average waveform of the task-related oxygenation process. Signal-averaging was applied to depict the task-related oxygenation response at each channel across all analyzed participants **(A)**. In the oxygenation process at the representative channel (channel 8), double-headed arrows indicate the target stimulus (S1) period, activation period, and the probe (query) stimulus (S2) period **(B)**. VWM, Verbal working memory; SWM, spatial working memory.

**Figure 5:**
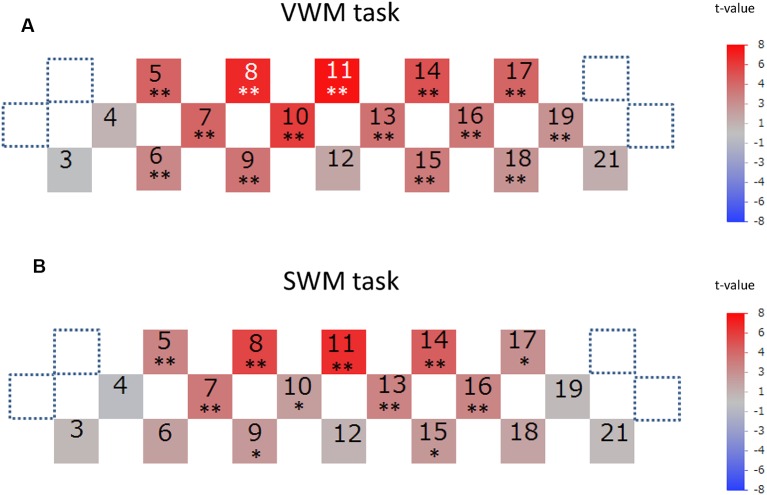
The significance of task-related prefrontal cortex (PFC) activation. Channels with significant task-related oxy-hemoglobin signal changes during the activation period at each channel across participants are illustrated by colored squares in the VWM task **(A)** and SWM task **(B)**. The color spectrum bars at the right end indicate the *t*-value scale. Channels with statistical significance are indicated by asterisks (*q < 0.05, **q < 0.01).

Signal-averaging of the task-related oxygenation process at each channel across all analyzed participants provided the grand average waveforms for each task (Figure 4). The delayed oxy-Hb signal peaks during the activation periods were observed at many channels in both tasks. The significance of these peaks as compared to the baseline of zero was statistically evaluated in each task (Figure 5). For the VWM task, the average task-related oxy-Hb signal was significantly increased in 14 channels (channels 5–11 and channels 13–19, *t* = 2.57–7.72, *p* ≤ 0.0094, *q* < 0.05; Figure 5A). The average SWM task-related oxy-Hb signal was significantly increased in 11 channels (channels 5, 7, 8, 9, 10, 11, and 13–17, *t* = 2.04–6.84, *p* ≤ 0.0280, *q* < 0.05; Figure 5B). These significant regions involved the bilateral DLPFC in both tasks. The raw data are available as supplementary data (Supplementary Tables S1, S2).

### Correlations Between the PFC Activity and Questionnaire Scores

**Figure 6:**
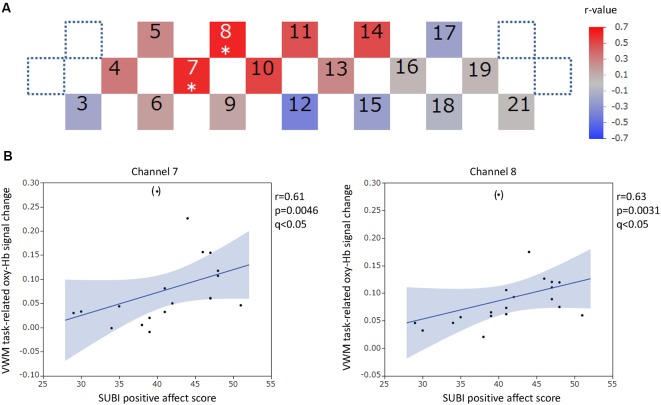
VWM task-related oxy-hemoglobin signal changes and the SUBI positive affect score. Levels of correlation are illustrated by colored squares **(A)**. The color spectrum bar indicates the *r*-value scale. Channels with statistical significance even after false discovery rate (FDR) correction are indicated by an asterisk (*q* < 0.05). Scatter plots with a regression line and 95% confidence interval represent the significant channels **(B)**. Outliers are indicated in parentheses. VWM, Verbal working memory; SUBI, Subjective Well-Being Inventory; oxy-Hb, oxy-hemoglobin.

**Figure 7:**
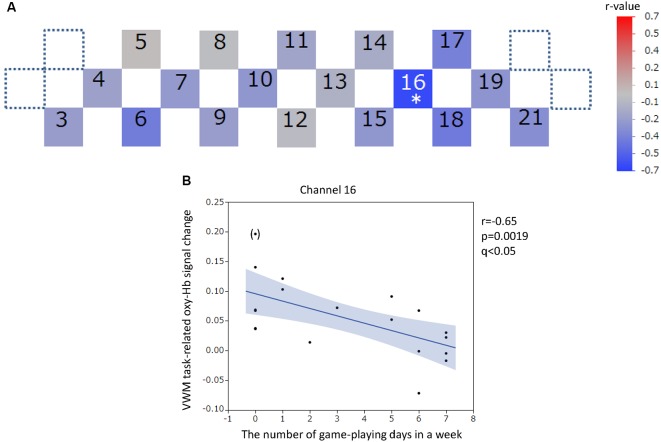
Verbal working memory task-related oxy-hemoglobin signal changes and the number of gaming days in 1 week. Levels of correlation are illustrated by colored squares **(A)**. The color spectrum bar indicates the *r*-value scale. The channel with statistical significance even after FDR correction is indicated by an asterisk (*q* < 0.05). The scatter plot with a regression line and 95% confidence interval represents the significant channel **(B)**. An outlier is indicated in parentheses. VWM, Verbal working memory; oxy-Hb, oxy-hemoglobin.

**Figure 8:**
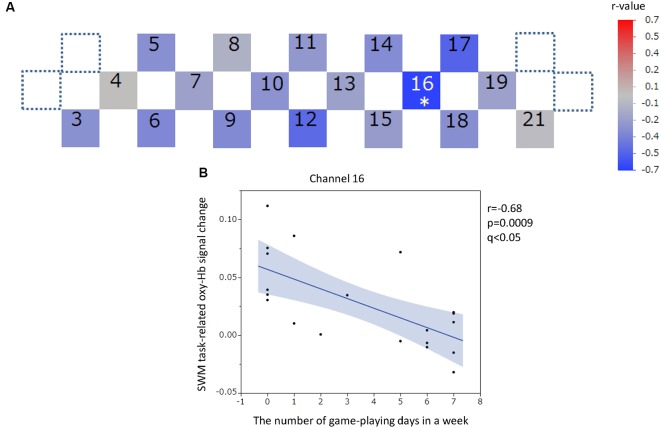
SWM task-related oxy-hemoglobin signal change and the number of gaming days in 1 week. Levels of correlation are illustrated by colored squares **(A)**. The color spectrum bar indicates the *r*-value scale. The channel with statistical significance even after FDR correction is indicated by an asterisk (*q* < 0.05). The scatter plot with a regression line and 95% confidence interval represents the significant channel **(B)**. SWM, Spatial working memory; oxy-Hb, oxy-hemoglobin.

**Figure 9:**
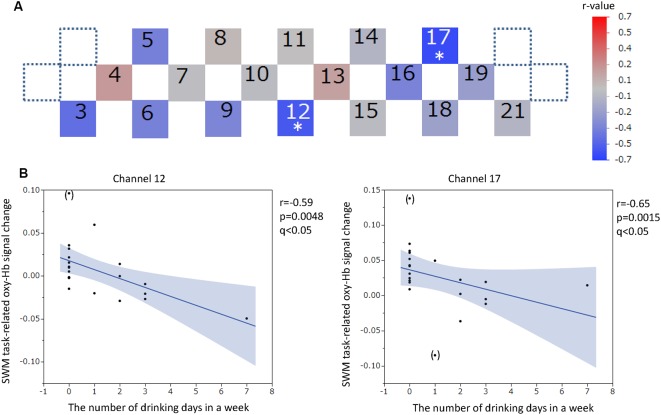
SWM task-related oxy-hemoglobin signal changes and the number of drinking days in 1 week. Levels of correlation are illustrated by colored squares **(A)**. The color spectrum bar indicates the *r*-value scale. Channels with statistical significance even after FDR correction are indicated by an asterisk (*q* < 0.05). Scatter plots with regression lines and 95% confidence intervals represent the significant channels **(B)**. Outliers are indicated in parentheses. SWM, Spatial working memory; oxy-Hb, oxy-hemoglobin.

The positions of channels that recorded task-related oxy-Hb signal changes that were significantly correlated with questionnaire scores are indicated in the channel array figure (Figures 6A, 7A, 8A, 9A). For the significant channels, the correlations are presented in scatter plots (Figures 6B, 7B, 8B, 9B).

#### VWM Task-Related Oxy-Hb Signal Changes and Questionnaire Scores

For the VWM task, significant positive correlations between the task-related oxy-Hb signal changes and SUBI positive affect scores were observed in two channels (channels 7 and 8) at the right DLPFC (channel 7: *r* = 0.61, *p* = 0.0046, *q* < 0.05; channel 8: *r* = 0.62, *p* = 0.0031, *q* < 0.05; Figure 6). Outlier removal provided more robust linear regressions in both channels (channel 7: *r* = 0.68, *p* = 0.0013; channel 8: *r* = 0.70, *p* = 0.0008). A significant negative correlation between oxy-Hb signal changes during the VWM task and the number of game playing days in 1 week was revealed in channel 16, at the left DLPFC (*r* = −0.65, *p* = 0.0019, *q* < 0.05; Figure 7). Outlier removal slightly undermined the significance (*r* = −0.62, *p* = 0.0047). Student’s *t*-test confirmed a significant difference in oxy-Hb signal change averages between students with game habits (*n* = 14) and those without game habits (*n* = 6; *t* = −2.0, *p* = 0.0286). The oxy-Hb signal changes during the VWM task did not significantly correlate with the SUBI negative affect score (*r* = −0.24 to 0.45), age (*r* = −0.18 to 0.36), number of drinking days in 1 week (*r* = −0.61 to −0.04), average internet use hours per day (*r* = −0.27 to 0.45), average smartphone use hours per day (*r* = −0.27 to 0.5), average game playing hours per day (*r* = −0.47 to 0.04), or number of days of exercise in 1 week (*r* = −0.35 to 0.44). In addition, there was no significant correlation between the VWM task-related PFC activation and task performance (accuracy: *r* = −0.22 to 0.32, reaction time: *r* = −0.19 to 0.58), and there was no significant difference between sexes in the VWM-task related oxy-Hb signal changes (*t* = −2.1 to 2.2).

#### SWM Task-Related Oxy-Hb Signal Changes and Questionnaire Scores

The oxy-Hb signal changes during the SWM task did not significantly correlate with the SUBI positive affect scores (*r* = −0.33 to 0.49) or SUBI negative affect scores (*r* = −0.51 to 0.60). A significant negative correlation between oxy-Hb signal changes during the SWM task and the average number of game playing days in 1 week was detected in channel 16, at the left DLPFC (*r* = −0.68, *p* = 0.0009, *q* < 0.05; Figure 8). This negative correlation (*n* = 20) was observed in the same channel as that in the VWM task (*n* = 20). A Student’s *t*-test confirmed a significant difference in oxy-Hb signal change averages between students with game habits (*n* = 14) and those without game habits (*n* = 7; *t* = −3.0, *p* = 0.0039). Significant negative correlations were also obtained between SWM task-related oxy-Hb signal changes and the number of drinking days in 1 week in channels 12 and 17, at the frontal pole (FP) and left DLPFC, respectively (channel 12: *r* = −0.59, *p* = 0.0048, *q* < 0.05; channel 17: *r* = −0.65, *p* = 0.0015, *q* < 0.05; Figure 9). In these two channels, outlier removal slightly undermined the significance (channel 12: *r* = −0.58, *p* = 0.0079; channel 17: *r* = −0.65, *p* = 0.0028). Student’s *t*-tests confirmed differences in oxy-Hb signal change averages between students with drinking habits (*n* = 9) and those without drinking habits (*n* = 12) in channels 12 (*t* = −2.0, *p* = 0.0308) and 17 (*t* = −3.2, *p* = 0.0024). On the other hand, in the relationship between drinking habits and PFC functions, the exclusion of participants without drinking habits (non-drinkers, *n* = 12) may have affected the correlation analysis. However, the negative correlation at channel 12 was not statistically significant in nine participants who had drinking habits (*r* = −0.62, *p* = 0.0726), and the negative correlation at channel 17 disappeared with removal of non-drinkers (*r* = 0.03, *p* = 0.9295). Additional outlier removal did not make the relationship significant at channel 17 (*r* = −0.30, *p* = 0.4698). The oxy-Hb signal changes during the SWM task did not significantly correlate with age (*r* = −0.18 to 0.36), the average internet use hours per day (*r* = −0.50 to 0.11), the average smartphone use hours per day (*r* = −0.56 to 0.36), the average game playing hours per day (*r* = −0.61 to −0.05), or the number of days of exercise in 1 week (*r* = −0.18 to 0.35). In addition, there was no significant correlation between the SWM task-related PFC activation and task performance (accuracy: *r* = −0.21 to 0.58; reaction time: *r* = −0.42 to 0.46), and there was no significant difference between sexes in the SWM-task related oxy-Hb signal changes (*t* = −0.41 to 2.46).

#### Partial Correlation Analysis

Three variables (the SUBI positive affect score, number of game playing days in 1 week, and number of drinking days in 1 week) that exhibited significant correlations with task-related oxy-Hb signal changes might influence each other with regard to their significance. In particular, there was a statistically significant correlation between gaming habits and drinking habits (Table 1). To evaluate possible complex interactions, partial correlation analyses were applied to analyze the interactions between task-related oxy-Hb signal changes and one of these three variables using the others as controls with the FDR threshold of 0.05. The regions of interest were channels 7, 8, 12, 16, and 17, at which a significant correlation was obtained more than once. As a result, the significant positive correlation between the VWM task-related PFC activation and SUBI positive affect scores was robust in channels 7 and 8, even after the elimination of the effects of the other variables (gaming and drinking habits; channel 7: *r* = 0.63, *p* = 0.0054, *q* < 0.05; channel 8: *r* = 0.6462, *p* = 0.0038, *q* < 0.05). The significant negative correlation between oxy-Hb signal changes during both working memory tasks and the number of game playing days in 1 week was also robust at channel 16, even after the elimination of the effects of the other variables (SUBI positive affect and drinking habit; VWM task: *r* = −0.61, *p* = 0.0034, *q* < 0.05; SWM task: *r* = −0.62, *p* = 0.0064, *q* < 0.05). However, the significant correlation between SWM task-related PFC activation and the number of drinking days in 1 week disappeared at both channel 12 and 17 after the elimination of the effects of the other variables (SUBI positive affect and gaming habit; channel 12: *r* = −0.43, *p* = 0.0695, *q* > 0.1; channel 17: *r* = −0.50, *p* = 0.0296, *q* > 0.1).

## Discussion

In the framework of the National Institute of Mental Health’s research domain criteria (RDoC), environmental factors provide the broad context and the RDoC matrix consists of the functional domains and their units of analysis (Clark et al., 2017). Despite several revisions of the operational diagnostic criteria over the past 50 years, our understanding of the interactions among these components and the roles of the RDoC matrix in mental disease manifestation or resilience is lacking (Clark et al., 2017). In the hierarchical complex underpinnings of mental conditions, subjective well-being can take an important position between the functional domains and disease manifestation (Burns et al., 2011). The aim of this study was to investigate the relationships between a unit of analysis (task-related PFC activation) and subjective well-being (SUBI scores) or environmental factors (lifestyle habits). In the VWM task, a statistically significant positive correlation was observed between PFC activity and the SUBI positive affect score and a statistically significant negative correlation was observed between PFC activity and the number of gaming days in 1 week. In the SWM task, a statistically significant negative correlation was observed between PFC activity and the number of gaming days in 1 week and a statistically significant negative correlation was observed between PFC activity and the number of drinking days in 1 week.

### Correlation Between Task-Related PFC Activation and the SUBI Positive Affect Score

In the integrative hierarchical model of depression and anxiety, each individual condition contains both a common and a unique underpinning (Mineka et al., 1998). Negative affectivity is shared by both conditions as a risk factor and low positive affect is related more specifically to depression in the model (Weinstock and Whisman, 2006; Burns et al., 2011). In the complex hierarchical structure, subjective well-being mediates the effects of psychological constructs on depression and anxiety (Burns et al., 2011). These possible roles on disease manifestation suggest the significance of subjective well-being as independent layer components in the hierarchical underpinnings of psychiatric conditions. In our study, a positive correlation between one of the affective measures of subjective well-being (SUBI positive affect) and VWM task-related oxy-Hb signal changes at the right DLPFC (channels 7 and 8) was statistically significant even after stringent correction (FDR). This correlation became more robust by excluding the outliers, and partial correlation analysis revealed that it was still robust in both channels in the absence of the confounding effects of lifestyle factors (gaming and drinking habits). Because the SUBI positive affect was inversely correlated with the level of negative mood (Tonan et al., 1995), which can be measured by a mood state questionnaire, the positive correlation in our results is concomitant with the reported significant negative correlation between VWM task-related PFC activities and premorbid levels of negative mood at the left DLPFC (Aoki et al., 2011). The negative correlation between task-related PFC activities and negative mood was demonstrated after the elimination of the impact of personality effects, particularly in the anterior PFC (Aoki et al., 2013), and was replicated in participants of different ethnicities mainly at the left DLPFC (Sato et al., 2014). Significant negative correlations were also revealed between VWM task-related PFC activities and changes (worsening) in negative mood at the left PFC (Sato et al., 2011). The possible differential relative lateralization in task-related PFC hemodynamic alterations by positive affect (right) and negative mood (left) may underline the significance of subjective well-being as an important emotional component in RDoC domains. In contrast to the results of the VWM task, there was no significant correlation between SWM task-related PFC activities and measures of subjective well-being (our results) or mood (Aoki et al., 2011, 2013; Sato et al., 2011, 2014), suggesting the relative predominance of word information processing in emotion-modifiable cognitive processes. Although functional magnetic resonance imaging (fMRI) indicates the bilateral activation of the DLPFC during working memory tasks (Braver et al., 2001), NIRS can detect the left-side dominance of VWM task-related PFC activities, particularly in women (Li et al., 2010; Gao et al., 2016). Men require higher engagement of the left PFC to perform verbal maintenance to achieve a similar performance to that of women (Gao et al., 2018). If both the significant modification of right PFC activities by SUBI positive affect and the significant modification of left PFC activities by negative mood contribute to the relative lateralization of verbal processing, the effects of subjective well-being might be observed *via* non-semantic information processes and those of mood might be observed *via* semantic processes. The possibility of a differential representation of semantic (left) emotion vs. non-semantic (right) emotion (Vrtička et al., 2013) may complement this argument. Although the possible lateralization or localization of emotional modification of verbal cognitive processes warrants further investigation, the presence of relative lateralization may indicate that such modification is an evolutionarily sustained function in a fundamental psychological module.

The positive correlation between VWM task-related PFC activities and measures of subjective well-being was previously suggested using a quality of life questionnaire in healthy participants (Satomura et al., 2014) and a subjective well-being scale in clinically stable patients with schizophrenia (Pu et al., 2013). Our findings regarding positive affect by the differential assessment of affective measures of subjective well-being might have been predicted by the link between positive sensation state and increased task-related DLPFC activities. Although both positive and negative affect are significantly associated with psychiatric conditions and negative affect is remarkable in the early stage of stress-related psychiatric disease, the care required to improve positive affect is eventually critical and positive affect evaluation is highly important for social recovery (Ono et al., 1995). In primary prevention of psychiatric conditions, positive affect is related to stress coping mechanisms and resilience. Therefore, an accurate or objective evaluation of subjective positive affect is extremely important in the clinical setting. The suggested positive correlation between positive affect and VWM task-related hemodynamic changes at the right PFC in our study warrants further studies and highlights the possibility of differential objective evaluations of positive affect at the right PFC.

### Correlation Between Task-Related PFC Activation and the Number of Gaming Days in 1 Week

Statistically significant negative correlations between PFC activities during the VWM and SWM tasks and the number of gaming days in 1 week were revealed at the left DLPFC even after the FDR correction. The correlation during the VWM task was slightly undermined by outlier exclusion. Student’s *t*-tests confirmed differences in oxy-Hb signal change averages between students with game habits and those without game habits for both tasks. The correlations during both tasks were still robust after the elimination of the confounding effects of SUBI positive affect and drinking habit. In Baddeley’s model, working memory is conceptualized as a series of multiple components including two short-term storage systems; the phonological loop in the VWM and the visuospatial sketchpad in the SWM, whose common attentional control system is the central executive (Baddeley, 2010). The fourth component is the episodic buffer, which may combine multi-modal information and is observed *via* conscious awareness. In semantic information processing, the visuospatial sketchpad interacts with visual semantics (Baddeley, 2010). Because the visuospatial interactions (sketchpad and visual semantics) also use the left PFC to the same extent as the phonological loop and the right PFC (Bonner et al., 2016; Dores et al., 2017; Papagno et al., 2017), the negative correlations at the left DLPFC between VWM and SWM task-related PFC activities and gaming habit may suggest the presence of modality-nonspecific hemodynamic influences by habitual gaming. In Baddeley’s model, modality-non-specific regulation can occur *via* effects on the central executive, episodic buffer, or nonspecific arousal level. These results were in line with earlier NIRS studies reporting that the PFC oxy-Hb decreased during videogame playing in healthy participants (Matsuda and Hiraki, 2005, 2006). In fMRI studies, reward-related decreases in neuronal activation in the DLPFC were demonstrated after training with a commercial videogame (Gleich et al., 2017) or during a complex visuomotor task (Lee et al., 2012). Because the blood-oxygenation level dependent (BOLD) neuronal activity in fMRI can be influenced by cerebral blood flow, the reported decrease in BOLD activities may include a blood flow change at the DLPFC. One single photon emission computed tomography (SPECT) study revealed that cerebral blood flow in young adults was significantly decreased in the PFC (mainly left) after videogame playing (Chou et al., 2013). The game-related alteration of task-induced hemodynamic changes in the left DLPFC, which was suggested in our study, may be associated with game-related PFC hypo-perfusion. Because the game-related PFC hypo-perfusion is observed both during and immediately after game playing (Lee et al., 2012; Chou et al., 2013), the delayed development theory (Takeuchi et al., 2016) cannot account for these findings. In the fMRI findings, the reduction in brain activation (possible hypo-perfusion) was correlated with improved task performance (Lee et al., 2012; Gleich et al., 2017). Replication of these findings in association with the “neuronal efficiency” hypothesis (Grabner et al., 2004; Gao et al., 2018), which predicts that lower cortical activation reflects higher neural efficiency, may be required.

“Internet gaming disorder” is included in section III of the fifth edition of Diagnostic and Statistical Manual of Mental Disorders and “Gaming disorder” will be included in the International Classification of Disease-11 (ICD-11; Higuchi et al., 2017). Furthermore, excessive game playing is associated with manifestation of other psychotic symptoms (Griffiths and Meredith, 2009; Petry et al., 2015; Stockdale and Coyne, 2018). To investigate the relationship between gaming disorders and other psychotic symptoms and elucidate the neuronal basis of gaming habit-related changes, task-related PFC hemodynamic changes using wearable NIRS may provide a useful unit of analysis because of the fewer limitations in the experimental environment relative to those associated with other functional neuroimaging methods.

### Correlation Between Task-Related PFC Activation and Number of Drinking Days in a Week

Statistically significant negative correlations between SWM task-related PFC activities and the number of drinking days in 1 week were observed at the FP and left DLPFC even after the FDR correction. Student’s *t*-tests confirmed differences in oxy-Hb signal change averages between students with drinking habits and those without drinking habits in both channels. Outlier removal slightly undermined the significance and the removal of non-drinkers made these correlations statistically non-significant. Although it is important to consider the effects of alcohol consumption on PFC functions because of the reported disadvantages of drinking alcohol (Pfefferbaum et al., 2001; Oscar-Berman and Marinković, 2007; Squeglia et al., 2011), further studies are needed to confirm these results.

### Limitations

The traditional concerns regarding the effects of task-related changes in the skin blood flow and anatomical factors including individual scalp-cortex distance (Hoshi et al., 2005; Haeussinger et al., 2011; Takahashi et al., 2011; Heinzel et al., 2013) have already been solved by seminal studies by authorities using simultaneous NIRS and fMRI measurement (Sato et al., 2013). They concluded that the impact of the skin blood flow and variation in the optical path length was sufficiently small, particularly in the DLPFC.

One limitation of the study design in the correlation analysis between gaming habit and task-related PFC activation should be addressed; our design did not segregate types of games. Brain hemodynamic activities varied depending on the type of videogame and the impact on neural mechanisms, and mental health might differ between those who play internet games and those who play offline games (Petry et al., 2015). One SPECT study demonstrated a violent game-specific cerebral blood flow change after videogame playing (Chou et al., 2013). Subtypes of gaming disorders that are classified by the predominance of online or offline gaming will be included in the ICD-11 (Király and Demetrovics, 2017).

In this study, participants were all Japanese students who attended the same university. Socioeconomic and demographic factors, including age, education, intellectual level, academic activities, and lifestyle details (the details of smartphone use and types of games) were not evaluated in our investigation. Further analyses with larger sample sizes and more detailed considerations of various confounding factors are warranted to ensure the significance and implications of our results, particularly those associated with individual well-being. In order to investigate the contribution of trait variables, developmental trends, twin-studies, and consideration of psychopathology or personality variables may be important. The study of patients with mental illness may be required to investigate the influence of subjective ill-being.

## Conclusion

In this study of university students with relatively similar backgrounds, we demonstrated that PFC hemodynamic activities during working memory tasks might be modifiable by positive affect (as assessed by the SUBI) and lifestyle habits, which may impact mental health. Although further replication studies are warranted to confirm these preliminary results, subsequent and more detailed investigations of the relationships between task-related hemodynamic activities and emotional domains (i.e., well-being) or lifestyle habits may contribute to the elucidation of the neural mechanism concerning vulnerability and resilience to mental illness and neural processes of disease manifestation. Because the wearable NIRS device can provide a quiet, non-invasive, and low-constraint experimental environment, and does not require any additional equipment or a large space, it is easy to use in schools or workplaces, where primary prevention is an important topic of interest.

## Data Availability

The raw data supporting the conclusions of this manuscript will be made available by the authors, without undue reservation, to any qualified researcher.

## Ethics Statement

This study was approved by the Institutional Review Board Ethics Committee of the Health Service Center, Kagoshima University (accession no.: H26-4-1). Written informed consent was obtained from all participants after the purpose, procedure, risks, benefits, personal information management, and voluntary nature of participation had been explained to them. In the analysis processes, all data were anonymized and the researchers were blinded to the links between the data and personal information.

## Author Contributions

YK designed and performed the experiment, analyzed the data, and wrote the manuscript. JN administered the SUBI and lifestyle questionnaire, and contributed to NIRS measurement. TF created the figures and performed analyses to double-check the results. CK provided technical advice regarding statistical analyses. MN performed Wilcoxon rank sum test and contributed to the manuscript revision. AS contributed to the manuscript revision. All authors have read and approved this manuscript.

## Conflict of Interest Statement

The authors declare that the research was conducted in the absence of any commercial or financial relationships that could be construed as a potential conflict of interest.
