# Supplementary material for: Working Memory-Related Prefrontal Hemodynamic Responses in University Students: A Correlation Study of Subjective Well-Being and Lifestyle Habits
Source: Front Behav Neurosci. 2019 Sep 13;13:213. doi: 10.3389/fnbeh.2019.00213 (PMC6754075; doi:10.3389/fnbeh.2019.00213)
Supplement: Supplementary file 1 [file Data_Sheet_1.docx]

Supplementary Material

Working memory-related prefrontal hemodynamic responses in university students: a correlation study with subjective well-being and lifestyles

Yoichi Kawaike*, Junko Nagata, Tamotsu Furuya, Chihaya Koriyama, Masayuki Nakamura, Akira Sano

*** Correspondence:** Yoichi Kawaike: oyatto@hsc.kagoshima-u.ac.jp

# Supplementary Data

Table S1

Task-related oxy-Hb signal change for VWM task

| Participant | CH3 | CH4 | CH5 | CH6 | CH7 | CH8 | CH9 | CH10 | CH11 | CH12 | CH13 | CH14 | CH15 | CH16 | CH17 | CH18 | CH19 | CH21 |
| --- | --- | --- | --- | --- | --- | --- | --- | --- | --- | --- | --- | --- | --- | --- | --- | --- | --- | --- |
| P1 | 0.03893 | 0.10762 | 0.191087 | 0.082094 | 0.22615 | 0.174688 | 0.040438 | 0.04598 | 0.120102 | -0.0053 | 0.135421 | 0.218731 | 0.033432 | 0.102936 | 0.093453 | 0.013674 | 0.078736 | 0.050145 |
| P2 | 0.03791 | 0.026414 | 0.077273 | -0.01491 | 0.050179 | 0.093138 | -0.03213 | 0.080191 | 0.104821 | -0.00511 | 0.002868 | 0.07806 | -0.03153 | 0.067197 | 0.088785 | 0.046385 | 0.125526 | 0.053037 |
| P3 | -0.01392 | -0.0214 | 0.025346 | 0.031199 | 0.04412 | 0.056489 | 0.065012 | 0.043958 | 0.049116 | 0.017405 | 0.015952 | 0.04419 | 0.038119 | 0.036563 | 0.049327 | 0.028919 | -0.00179 | -0.02526 |
| P4 | 0.036191 | 0.020071 | 0.07963 | 0.060386 | -0.00889 | 0.058793 | 0.02338 | 0.015684 | 0.031255 | 0.00336 | -0.01483 | 0.077762 | 0.015063 | -0.01698 | 0.091344 | 0.015773 | 0.084428 | -0.02556 |
| P5 | 0.006068 | -0.02154 | 0.024141 | 0.023115 | 0.030282 | 0.046061 | 0.057507 | 0.041044 | 0.051222 | 0.063086 | 0.041319 | 0.052251 | 0.085546 | 0.068574 | 0.05161 | 0.054297 | 0.051874 | 0.036732 |
| P6 |  | -0.03872 | 0.039777 | 0.012484 | 0.061171 | 0.110387 | 0.030194 | 0.062456 | 0.081719 | 0.029451 | 0.091859 | 0.083501 | 0.027492 | 0.091066 | 0.086724 | 0.023906 | 0.053191 | -0.02914 |
| P7 | -0.01391 |  | 0.017003 | 0.064908 | 0.154934 | 0.120378 | 0.084789 | 0.044773 |  | 0.004997 |  |  | 0.031551 | -0.00107 | 0.01368 | 0.048435 | 0.109304 | 0.060893 |
| P8 | 0.003519 | 0.00738 | 0.057059 | 0.000936 | 0.081219 | 0.105656 | 0.038273 | 0.040098 | 0.07472 | 0.031883 | 0.11847 | 0.0553 | 0.04577 | -0.00468 | 0.007501 | -0.01363 | -0.03321 | -0.04375 |
| P9 | -0.01688 | 0.015363 | 0.052404 | 0.007564 | 0.005545 | 0.020835 | -0.02464 | -0.01616 | 0.014769 | -0.03501 | 0.015322 | 0.039032 | 0.056462 | 0.067289 | 0.009162 | 0.022089 | -0.01481 | -0.00782 |
| P10 | -0.001 | 0.018898 | 0.034872 | -0.00623 | -0.00101 | 0.046178 | -0.00414 | 0.015265 | 0.069586 | 0.012308 | 0.037777 | 0.071739 | 0.014165 | 0.013902 | 0.032247 | 0.000101 | 0.009283 | 0.023069 |
| P11 | -0.00547 | 0.017475 | 0.058576 | -0.012 | 0.020209 | 0.065378 | -0.00477 | 0.012578 | 0.05493 | -0.01935 | 0.037394 | 0.065187 | 0.004581 | 0.030218 | 0.022963 | -0.00225 | -0.01742 | 0 |
| P12 | -0.00759 | 0.057778 | 0.076192 | 0.026856 | 0.081222 | 0.073388 | 0.052818 | 0.045386 | 0.051427 | 0.006443 | 0.031062 | 0.071661 | 0.052519 | 0.072062 | 0.073762 | 0.080031 | 0.085125 | 0.021904 |
| P13 | -0.00449 | -0.00814 | 0.146036 | 0.088181 | 0.156355 | 0.126359 | 0.085231 | 0.087642 | 0.147468 | -0.00187 | 0.198077 | 0.172949 | 0.13166 | 0.121067 | 0.16755 | 0.132829 | 0.072341 | 0.028499 |
| P14 | 0.006213 | -0.01906 | 0.030161 | 0.015356 | 0.033293 | 0.03239 | 0.002967 | 0.006791 | 0.024657 | 0.037651 | 0.022446 | 0.033908 | 0.029451 | 0.052085 | 0.030732 | 0.017394 | 0.025806 | -0.03597 |
| P15 | 0.046068 | 0.032005 | 0.32167 | 0.073466 | 0.281812 | 0.278503 | 0.080318 | 0.118269 | 0.143205 | 0.064805 | 0.176155 | 0.318277 | 0.115758 | 0.196189 | 0.163071 | 0.065647 | 0.140902 | 0.12209 |
| P16 | 0.005374 | 0.022418 | 0.100174 | 0.008639 | 0.1177 | 0.119561 | 0.039104 | 0.101762 | 0.122206 | -0.00453 | 0.078333 | 0.10383 | 0.022326 | 0.022052 | 0.017882 | 0.008935 | 0.00816 | 0.006543 |
| P17 | -0.01174 | 0.032395 | 0.06179 | 0.023368 | 0.107204 | 0.075093 | -0.01213 | 0.042339 | 0.056363 | -0.03646 | 0.057083 | 0.074316 | 0.018101 | 0.140236 | 0.029559 | -0.04158 | 0.038131 | -0.07305 |
| P18 | -0.01733 | 0.024316 | 0.049256 | 0.002143 | 0.032439 | 0.062006 | 0.020485 | 0.03702 | 0.026098 | 0.005028 | -0.00512 | 0.016156 | -0.01208 | 0.022153 | 0.049024 | 0.039134 | 0.074012 | 0.034451 |
| P19 | -0.00458 | 0.036535 | 0.039669 | 0.078762 | 0.046166 | 0.059852 | 0.041533 | 0.03633 | 0.041895 | -0.017 | -0.00403 | 0.053355 | 0.021773 | 0.037518 | 0.007739 | 0.040542 | -0.0014 | 0.034171 |
| P20 | -0.10013 | -0.14075 | 0.035805 | -0.04655 | 0.060499 | 0.08917 | 0.042774 | 0.086271 | 0.092559 | 0.048992 | 0.077968 | 0.097016 | -0.02249 | -0.07167 | -0.0298 | -0.07605 | -0.13596 | -0.00186 |

Note : Blank represent missing value. Zero was resulted as the activation values in all blocks were zero.

Table S2

Task-related oxy-Hb signal change for SWM task

| Participant | CH3 | CH4 | CH5 | CH6 | CH7 | CH8 | CH9 | CH10 | CH11 | CH12 | CH13 | CH14 | CH15 | CH16 | CH17 | CH18 | CH19 | CH21 |
| --- | --- | --- | --- | --- | --- | --- | --- | --- | --- | --- | --- | --- | --- | --- | --- | --- | --- | --- |
| P1 | 0.01743 | 0.064597 | 0.190077 | 0.061698 | 0.205936 | 0.169534 | 0.020475 | 0.028681 | 0.105187 | -0.00211 | 0.115696 | 0.246984 | 0.001383 | 0.085827 | 0.063313 | 0.019601 | 0.083772 |  |
| P2 | 0.054193 | 0.072005 | 0.062333 | 0.033211 | 0.053755 | 0.040873 | 0.033851 | 0.000123 | 0.061516 | -0.00152 | -0.0661 | 0.042211 | -0.01773 | 0.070492 | 0.073343 | 0.019586 | 0.060642 | 0.029465 |
| P3 | 0.017126 | -0.01529 | 0.028096 | 0.014402 | -0.0145 | 0.014349 | 0.043609 | -0.01179 | 0.041494 | 0.015485 | 0.028486 | 0.024773 | 0.024991 | 0.030395 | 0.021106 | 0.046621 | 0.026713 | 0.005088 |
| P4 | 0.019331 | -0.00061 | 0.061384 | 0.02881 | 0.026367 | 0.026163 | 0.025876 | 0.000581 | 0.00073 | -0.00244 | -0.03009 | 0.033905 | -0.00017 | 0.011281 | 0.051818 | -0.0117 | 0.016272 | -0.01528 |
| P5 | 0.021455 | 0.015527 | 0.028547 | 0.023989 | 0.062755 | 0.038201 | 0.057485 | 0.058009 | 0.053827 | 0.059408 | 0.050242 | 0.054766 | 0.092604 | 0.075395 | 0.049374 | 0.053027 | 0.053273 | 0.029468 |
| P6 |  | -0.05788 | 0.030579 | -0.01964 | 0.031086 | 0.100279 | -0.00598 | 0.019459 | 0.0421 | 0.005076 | 0.011502 | 0.017476 | -0.00666 | -0.00509 | 0.008716 | -0.0042 | -0.0159 | -0.04831 |
| P7 | 0.008135 |  | -0.10628 | 0.031262 | 0.059711 | 0.02632 | 0.042092 | -0.03433 | 0 | -0.00031 |  |  | 0.03713 | 0.004269 | -0.03668 | 0.056952 | 0.036033 | 0.035012 |
| P8 | -0.0254 | -0.01703 | 0.006503 | -0.01242 | 0.082533 | 0.099781 | 0.032027 | 0.029726 | 0.075285 | -0.0269 | 0.118483 | 0.047606 | 0.0434 | -0.03192 | -0.01205 | 0.018653 | -0.0275 | -0.01991 |
| P9 | -9.4E-06 | 0.026073 | -0.01383 | -0.00138 | -0.01211 | -0.00803 | -0.01295 | -0.01135 | -0.00302 | -0.00948 | -0.01477 | 0.002638 | 0.00127 | -0.00662 | -0.00528 | -0.00845 | 0.008482 | -0.03393 |
| P10 | -0.02095 | -0.01186 | 0.027347 | -0.02093 | -0.01041 | 0.039952 | -0.01497 | -0.00657 | 0.052763 | 0.010611 | 0.024843 | 0.037484 | -0.00328 | 0.000699 | 0.024532 | -0.01504 | 0.004799 | 0.013406 |
| P11 | -0.03281 | -0.00275 | 0.032492 | -0.01696 | 0.005838 | 0.042039 | -0.01245 | -0.01199 | 0.035156 | -0.02906 | 0.021626 | 0.045207 | 0.009124 | 0.019792 | 0.002121 | -0.00112 | -0.01628 | 0 |
| P12 | 0.03382 | 0.033411 | 0.032979 | 0.026267 | 0.040437 | 0.035226 | 0.055202 | 0.019798 | 0.029042 | 0.035564 | 0.01454 | 0.034166 | 0.046457 | 0.034604 | 0.041636 | 0.035318 | 0.042344 | 0.037446 |
| P13 | -0.00462 | -0.02529 | 0.048724 | 0.023942 | 0.033525 | 0.000355 | 0.032723 | 0.014631 | 0.022323 | 0.031604 | 0.012888 | 0.014962 | 0.012557 | 0.010219 | 0.030922 | 0.048706 | 0.048201 | 0.027028 |
| P14 | -0.00088 | -0.02871 | 0.028603 | 0.001406 | 0.027319 | 0.035796 | 0.013424 | 0.00209 | 0.022638 | 0.009688 | 0.02258 | 0.050374 | 0.010925 | 0.071749 | 0.042787 | 0.012308 | 0.01322 | -0.02101 |
| P15 | 0.01172 | -0.05354 | 0.07862 | 0.001989 | 0.071229 | 0.111894 | 0.00535 | 0.047244 | 0.070016 | 0.021516 | 0.042403 | 0.140821 | 0.018614 | 0.039295 | 0.01853 | -0.03519 | -0.07364 | 0.129018 |
| P16 | 0.042731 | 0.025879 | 0.109325 | 0.030206 | 0.101949 | 0.127853 | 0.045086 | 0.102214 | 0.131806 | 0.013838 | 0.076456 | 0.119137 | 0.027303 | 0.018809 | 0.022198 | 0.006596 | -0.00442 | 0.045286 |
| P17 | -0.03773 | -0.00193 | 0.045267 | -0.00841 | 0.05063 | 0.10329 | -0.02084 | 0.032544 | 0.066852 | -0.02077 | 0.084209 | 0.102377 | 0.004978 | 0.111709 | 0.019153 | -0.02737 | -0.01597 | -0.0963 |
| P18 | -0.03651 | 0.006117 | 0.032121 | -0.02835 | 0.016273 | 0.043328 | -0.01678 | 0.004099 | 0.016933 | -0.0495 | 0.004744 | 0.007697 | -0.04946 | -0.01504 | 0.01438 | 0.003936 | 0.039118 | 0.029373 |
| P19 | -0.00161 | -0.02802 | 0.037955 | 0.091696 | 0.062686 | 0.110147 | 0.054207 | 0.0753 | 0.08841 | -0.015 | 0.035876 | 0.107269 | 0.027569 | 0.035086 | 0.060733 | 0.045212 | -0.01734 | 0.024111 |
| P20 | -0.04347 | -0.05732 | -0.01704 | -0.01015 | -0.02285 | 0.045702 | -0.02427 | -0.03842 | 0.074398 | -0.0202 | 0.050924 | 0.070531 | -0.00181 | -0.01031 | -0.08502 | -0.06685 | -0.15279 | -0.04819 |
| P21 | 0.050441 |  | 0.114324 |  |  | 0.055189 | 0.252119 |  | 0.049677 | 0.096022 | 0.089538 | 0.073889 | 0.145819 |  | 0.13792 | 0.108102 |  | -0.0378 |

Note : Blank represent missing value. Zero was resulted as the activation values in all blocks were zero.

**
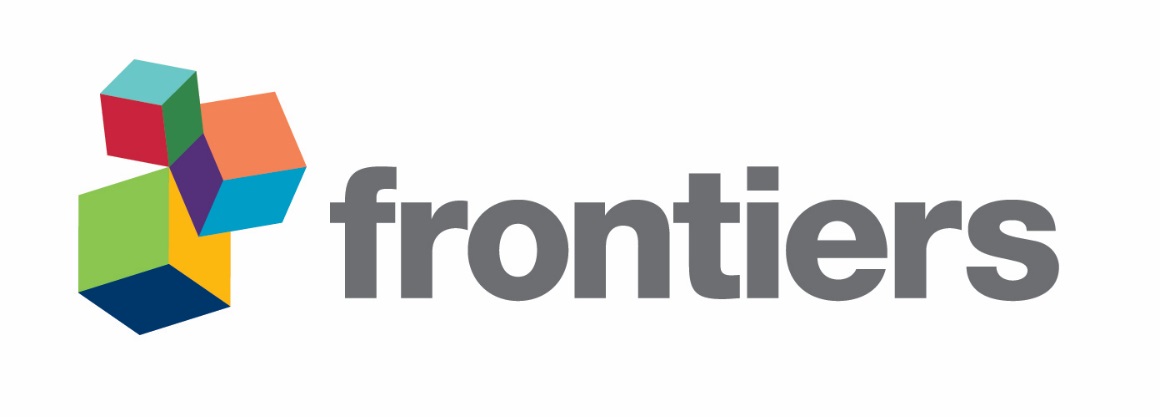
**
